# Valvular and aortic surgery in an adult patient late after repaired tetralogy of Fallot

**DOI:** 10.1186/s44215-022-00016-z

**Published:** 2022-12-08

**Authors:** Ryuya Nomura, Kojiro Furukawa, Tomofumi Fukuda, Yuichiro Hirata, Tatsushi Onzuka, Kisho Ohtani, Ken-ichi Imasaka, Shigeki Morita, Akira Shiose

**Affiliations:** 1 Department of Cardiovascular Surgery, Clinical Research Institute, National Hospital Organization Kyushu Medical Center, Fukuoka, 810-8563 Japan; 2 Department of Surgery II, University of the Ryukyus, Okinawa, Japan; 3 Department of Cardiology, Clinical Research Institute, National Hospital Organization Kyushu Medical Center, Fukuoka, Japan; 4 Department of Cardiovascular Surgery, Kyushu University Hospital, Fukuoka, Japan

**Keywords:** Tetralogy of Fallot, Pulmonary valve, Tricuspid valve, Aorta, Adult congenital heart disease

## Abstract

**Background:**

The most common complication after tetralogy of Fallot repair is pulmonary valve regurgitation. However, concomitant surgery for tricuspid valve regurgitation and/or aortic dilatation is occasionally required.

**Case presentation:**

A 56-year-old woman who underwent surgery for tetralogy of Fallot at age 29 years was diagnosed with progressive tricuspid valve regurgitation, hepatosplenomegaly, and thrombocytopenia. Moreover, computed tomography and transesophageal echocardiography revealed regurgitation in pulmonary and mitral valves and dilatation of the ascending aorta. One month after splenectomy for increasing platelet count, she underwent pulmonary valve replacement, tricuspid and mitral valve annuloplasty, and ascending aortic replacement. Postoperatively, the intensive care stay was complicated because of ventricular dysfunction and long-term ventilation. After 1 week, the patient was disconnected from the respirator, and she was transferred on the 55th postoperative day to another hospital for rehabilitation, without permanent disabilities.

**Conclusion:**

We herein reported a patient with repaired TOF who successfully underwent PVR, tricuspid and mitral valve annuloplasty, and ascending aortic replacement.

## Introduction

Tetralogy of Fallot (TOF) is the most prevalent cyanotic congenital heart disease. While the current early survival associated with total repair of TOF is excellent, and the reported mortality rate was only 0.8% in Japan [1], some studies of adult survivors with repaired TOF showed several late complications, namely pulmonary valve regurgitation (PR), tricuspid valve regurgitation (TR), atrial tachyarrhythmia, and aortic dilation. Long-term PR carries the risk of progressive right ventricular dilation and dysfunction and secondary TR. Pulmonary valve replacement (PVR) is a frequent surgical procedure in the right ventricular outflow tract, but optimal timing for PVR and surgical management of TR, which is observed in up to 30% of patients with repaired TOF, remain controversial [2, 3]. Atrial tachyarrhythmia, which is observed in approximately one-third of repaired TOF patients, can cause atrial functional mitral valve regurgitation [2, 4]. In addition, aortic root and ascending aortic dilation exceeding 40 mm after TOF repair are reported in 15–28.9% of patients, which may be ascribable to an intrinsic mechanism or a secondary effect of increased volume overload of the aorta caused by right-to-left shunting [5, 6].

We herein describe a rare case of a 56-year-old woman with repaired TOF who had severe PR and TR, moderate mitral valve regurgitation, and ascending aortic dilation. She underwent successful PVR, tricuspid and mitral valve annuloplasty, and ascending aortic replacement.

## Case

**Fig. 1:**
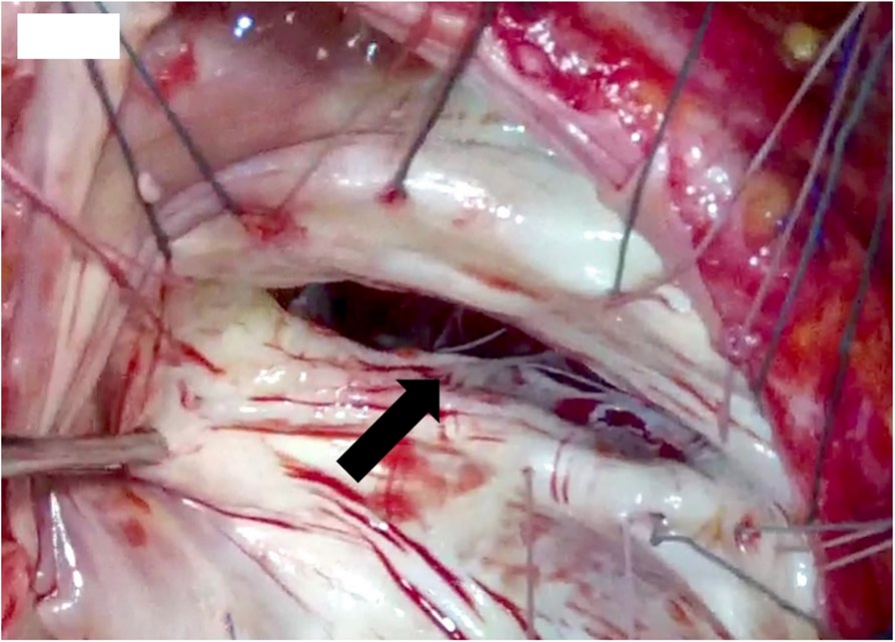
Intraoperative findings of tricuspid valve. The annulus was remarkably dilated, but there was no chordal disruption or septal-leaflet (black arrow) distortion secondary to ventricular septal defect closure at the time of complete tetralogy of Fallot repair

**Fig. 2:**
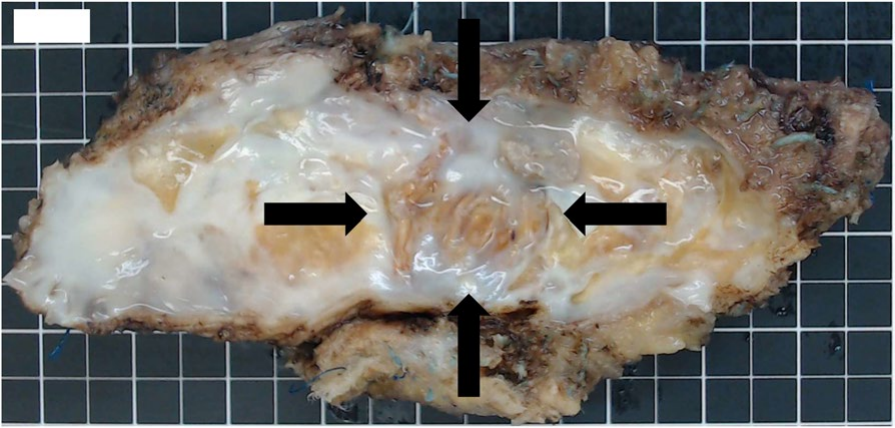
The debrided MVOP. The patch was totally and remarkably calcified, and its mono-cusp (black arrows) did not work at all because of severe adhesion to the patch

The patient was a 56-year-old woman diagnosed with TOF after birth. She underwent a left classical Blalock-Taussig shunt at age 5 years, complete repair using mono-cusp ventricular outflow patch (MVOP) at age 29 years (there were no detailed records about these surgical treatments), and catheter ablation for atrial flutter at age 44 years. She was diagnosed with atrial fibrillation and moderate TR at 48 years of age and reduced ejection fraction (left ventricular ejection fraction: 32.4%), progressive TR, and hepatosplenomegaly with exercise intolerance, and thrombocytopenia by follow-up transthoracic and abdominal ultrasonography was found at 55 years of age. Transesophageal echocardiography showed severe PR owing to MVOP failure, severe TR owing to tricuspid annular dilation (tricuspid annulus diameter index: 40.7 mm/m^2^), moderate mitral valve regurgitation owing to permanent atrial fibrillation, and mild aortic valve regurgitation owing to ascending aortic dilation. Right ventricular end-diastolic volume index (RVEDVI) and pulmonary regurgitation fraction (PRF) using cardiac magnetic resonance (CMR) were 129.2 ml/m^2^ and 52.0%, respectively. Computed tomography revealed a dilated ascending aorta (maximum diameter: 55.0 mm) and hepatosplenomegaly owing to congestive heart failure and hepatopathy. The multiple valve and aortic surgery increased operative risk in this case (EuroSCORE II: 37.4%). Furthermore, the patient had thrombocytopenia secondary to hypersplenism; therefore, hand-assisted laparoscopic splenectomy was performed first, and 1 month later, her platelets increased from 5.0 × 10^4^/μl to 29.4 × 10^4^/μl. She subsequently underwent PVR, tricuspid valve annuloplasty, mitral valve annuloplasty, and ascending aortic replacement. A full median sternotomy was performed. Cardiopulmonary bypass was established using bilateral femoral artery, right femoral vein, and superior vena cava cannulation. First, ascending aortic replacement above the sinotubular junction (J-graft, 28 mm; Japan Lifeline, Tokyo, Japan) was performed under hypothermic circulatory arrest with selective cerebral perfusion. Second, following systemic reperfusion with cardiac arrest, mitral valve annuloplasty (Physio II, 28 mm; Edwards Lifesciences, Irvine, CA) was performed via the transseptal approach. Third, tricuspid valve annuloplasty (Physio tricuspid ring, 26 mm; Edwards Lifesciences) was performed. The cause of the TR was tricuspid annular dilation with intact leaflets owing to right ventricular dilatation (Fig. 1). Finally, PVR (INSPIRIS RESILIA, 23 mm; Edwards Lifesciences) was performed. A longitudinal incision was made in the proximal outflow tract, and all calcifications, including of the MVOP (Fig. 2), were debrided as appropriate. Then, the stented bioprosthetic valve was sewn into the orthotopic position in the right ventricular outflow tract (operation time: 764 min, cardiopulmonary bypass time: 401 min, cardiac arrest time: 280 min, lower body ischemia time: 39 min). After the operation, the intensive care stay was complicated owing to ventricular dysfunction and long-term ventilation. One week later, she was disconnected from the respirator and was transferred on postoperative day 55 to another hospital for rehabilitation, without permanent disabilities. Although the postoperative ECG findings was still atrial fibrillation, transthoracic echocardiography showed good function of the prosthetic pulmonary valve, only mild TR, and trivial mitral and aortic valve regurgitation.

## Discussion

The causes of late reoperation after TOF repair are PR, TR, atrial tachyarrhythmia, and a dilated aorta. We performed PVR, mitral and tricuspid valve annuloplasty, and ascending aortic replacement 27 years after complete TOF repair, in this case.

Chronic PR is a frequent consequence of repaired TOF (especially with transannular patches) and results in right ventricular dilation and dysfunction, exercise intolerance, arrhythmia, and sudden cardiac death. However, the timing of PVR remains controversial. Tatewaki et al. suggested exercise intolerance, arrhythmia, *PRF* > 40% by CMR, and *RVEDVI* > 150 ml/m^2^ by CMR as criteria for PVR [2]. In our case, the patient experienced a good clinical course after surgery, but it is possible that PVR should have been performed earlier because she had preoperative severe PR (*PRF*: 52.0%) with exercise intolerance, permanent atrial fibrillation, and congestive hepatopathy owing to congestive heart failure.

Functional TR in adults with repaired TOF is common and mainly results from tricuspid annular dilation caused by right ventricular dilation secondary to long-term PR. In some studies, PVR alone in repaired TOF with TR led to significant improvement in TR grade, tricuspid valve annulus diameter, and right ventricular volume without concomitant tricuspid valve surgery [7, 8]. In contrast, in a study by Roubertie et al., the authors suggested that in cases of severe TR, tricuspid valve repair should be considered at the time of PVR because postoperative moderate or greater TR might remain after PVR without tricuspid valve repair. In addition, the authors introduced two mechanisms of TR: (1) a functional etiology, such as annulus dilation, and (2) an iatrogenic etiology owing to chordal disruption or tricuspid septal-leaflet distortion secondary to ventricular septal defect closure at the time of the primary TOF repair. The authors suggested that annuloplasty should be performed in cases of isolated functional TR, while a partial closure of the anteroseptal commissure with annuloplasty should be performed for iatrogenic lesions [3]. Our patient had severe TR owing to a dilated tricuspid valve annulus without iatrogenic features (Fig. 1); therefore, she underwent successful concomitant tricuspid valve annuloplasty at the time of PVR.

Atrial tachyarrhythmia in repaired TOF patients is caused by two main circuits: (1) rotation along the edge of the tricuspid valve, with a narrow conduction corridor at the isthmus between the inferior vena cava and the tricuspid valve ring, and (2) rotation around a lateral atriotomy scar, with a narrow conduction corridor at the isthmus between the lower edge of the inferior vena cava. Deferm et al. reported that atrial tachyarrhythmia, such as atrial fibrillation, could cause atrial functional mitral valve regurgitation resulting from mitral valve annular dilation following left atrial dilation [4]. The authors also referred to annuloplasty as a treatment for atrial functional mitral valve regurgitation. Our patient had moderate mitral valve regurgitation resulting from permanent atrial fibrillation, and she underwent successful mitral valve annuloplasty.

Aortic root and ascending aortic dilation after TOF repair are attributable to an intrinsic mechanism, such as cystic medial necrosis, or a secondary effect of increased volume overload of the aorta because of right-to-left shunting. Reportedly, the risk factors for aortic dilation after repaired TOF are male sex, pulmonary atresia, right aortic arch, and longer time interval from palliation to repair [6, 9]. Aortic surgery should be considered in patients with an aortic diameter of ≥ 55 mm [9, 10]. In the current case, the patient’s ascending aorta dilated to 55 mm, but there was no cystic medial necrosis in the extracted ascending aorta tissue, intraoperatively. While TOF is often repaired in childhood, our patient underwent palliation at age 5 years and complete repair at age 29 years; therefore, the longtime interval could have caused aortic dilation. Although the patient had high EuroSCORE II and was a high surgical risk case, she was saved by prior splenectomy, cardiovascular surgery, and postoperative intensive care.

## Conclusion

We herein reported a case of repaired TOF in a patient who successfully underwent PVR, tricuspid and mitral valve annuloplasty, and ascending aortic replacement.

## Abbreviations

PR: Pulmonary valve regurgitation
TR: Tricuspid valve regurgitation
PVR: Pulmonary valve replacement
TOF: Tetralogy of Fallot
CMR: Cardiac magnetic resonance
MVOP: Mono-cusp ventricular outflow patch
RVEDVI: Right ventricular end-diastolic volume index
PRF: Pulmonary regurgitant fraction
